# The rhythmic mind: how music tempo shapes creative self-efficacy through cognitive flexibility

**DOI:** 10.3389/fpsyg.2026.1946328

**Published:** 2026-10-26

**Authors:** Jinjin Zhang, Dashan Meng

**Affiliations:** 1 College of Arts, Yanbian University, Yanji, China; 2 Research Center, Zhejiang Conservatory of Music, Hangzhou, China

**Keywords:** cognitive flexibility, creative self-efficacy, divergent thinking, music tempo, prior creative experience

## Abstract

**Background:**

Creative self-efficacy (CSE) is a critical predictor of creative achievement. While background music is ubiquitous in contemporary work and learning environments, the psychological mechanisms through which music tempo influences CSE remain poorly understood, and empirical findings have been inconsistent.

**Methods:**

A three-group between-subjects experimental design was employed. A total of 402 undergraduate students were randomly assigned to fast-tempo music, slow-tempo music, or no-music control conditions. Participants completed the Alternative Uses Task (AUT) while listening to their assigned auditory condition. Cognitive flexibility and CSE were measured immediately following the task. The moderated mediation model was tested using PROCESS Macro (Model 14) with 5,000 bootstrap resamples.

**Results:**

Fast-tempo music significantly enhanced cognitive flexibility compared to the silent control condition, whereas slow-tempo music did not. Cognitive flexibility significantly predicted CSE. The indirect effect of fast-tempo music on CSE through cognitive flexibility was significant. More importantly, prior creative experience moderated the flexibility-to-CSE pathway. The indirect effect was statistically significant for individuals with high prior creative experience, but not for those with low prior experience. In the supplementary AUT analyses, fast-tempo music was associated with higher fluency but not with higher originality.

**Conclusion:**

These findings reveal that fast-tempo music was associated with enhanced CSE indirectly through cognitive flexibility among individuals with a rich history of creative mastery. This dissociation suggests that fast tempo may be linked primarily to the quantity or speed of idea generation rather than to the novelty of the ideas produced. For those with low prior creative experience, the same auditory environment did not yield a statistically significant indirect effect; this should not be interpreted as evidence that the pathway is absent.

## 1 Introduction

Creativity is a multidimensional construct that has been conceptualized in diverse ways across artistic, scientific, and everyday domains. We rely specifically on Amabile’s (1996) definition of creativity as the capacity to produce ideas or outcomes that are both novel and useful, and it is widely regarded as one of the most valuable human attributes in artistic, scientific, and professional contexts. This ability underpins innovation, contributes to cultural evolution, and helps both individuals and organizations adapt to shifting circumstances (Fogarty et al., 2015; Ivcevic et al., 2024). Given its significance, a considerable body of empirical work has examined the factors that either promote or hinder creative performance (Cebrián and Ramos-Villagrasa, 2026). Within this broad research landscape, creative self-efficacy (CSE), understood as an individual’s belief in their own capacity to generate creative outputs, has been identified as a robust psychological antecedent of creative accomplishments (Haase et al., 2018; Tierney and Farmer, 2002). Beyond its descriptive role, CSE operates as a central motivational mechanism that influences how much effort, persistence, and resilience an individual is willing to commit when confronting ambiguous problems or potential failure (Chen et al., 2025; Liu et al., 2016; Tierney and Farmer, 2011).

Despite the well-established importance of CSE, relatively little is known about how routine environmental cues, particularly auditory inputs, might shape this CSE. Background music, which has become almost omnipresent in contemporary work and learning environments (de la Mora Velasco et al., 2023; Landay and Harms, 2019), represents a particularly compelling case in point. Music is frequently encountered during daily activities such as commuting, studying, working, and leisure; nonetheless, the psychological processes through which it influences creative confidence remain poorly specified. The existing evidence regarding music’s impact on creativity is mixed. Some studies indicate that background music can facilitate creative thinking by elevating positive mood and arousal (e.g., Ritter and Ferguson, 2017), whereas others report null or even negative effects, especially when the music includes vocal elements or carries a high degree of informational complexity (Threadgold et al., 2019; Perham and Currie, 2014). These discrepant outcomes have prompted scholars to advocate for a more nuanced perspective, one that moves away from treating music as a homogeneous stimulus and instead directs attention to its specific structural attributes and their interplay with individual differences (de la Mora Velasco et al., 2023).

Among the various structural elements of music, tempo, defined as the speed of music as measured in beats per minute (BPM), emerges as a particularly promising yet insufficiently examined factor. Although different rhythmic patterns can be performed at various tempi, tempo itself is a distinct structural parameter that can be manipulated independently of rhythm. This distinction is central to the present study, which holds rhythmic structure constant and varies tempo alone. Tempo is not merely a surface feature; it has a direct and fundamental bearing on human physiological and cognitive functioning. Specifically, tempo can entrain motor activity, modulate autonomic arousal (for instance, heart rate and cortisol secretion), synchronize oscillatory brain activity, and elicit robust affective responses (Honing, 2018; Thaut et al., 2015). From a cognitive neuroscience perspective, tempo cues influence temporal information processing, attentional allocation, and cognitive control through the entrainment of neural oscillations, particularly in the gamma and theta bands (Doelling and Poeppel, 2015; Large and Jones, 1999). This neural alignment between endogenous rhythms and external auditory inputs is thought to enhance the efficiency of information processing and to promote cognitive flexibility (Kenett et al., 2018). For example, a fast tempo tends to raise arousal levels and may facilitate rapid, associative idea production, which is characteristic of divergent thinking, whereas a slower tempo might encourage more deliberative, elaborative processing that supports convergent thinking or sustained reflection (De Francesco et al., 2025; Liu, 2026). Importantly, tempo can affect cognition without introducing the semantic interference commonly associated with lyrics, a confound that has complicated many prior investigations of music and creativity (Sun et al., 2024). By employing tempo as an isolated experimental manipulation, researchers can more precisely dissect its cognitive and emotional consequences.

Nevertheless, to fully grasp how tempo exposure translates into variations in creative self-efficacy, it is necessary to move beyond main-effect reasoning and consider the intermediate cognitive mechanisms through which auditory information shapes self-perception. Cognitive flexibility refers to the capacity to adaptively adjust one’s mental framework in response to changing environmental demands (Dennis and Vander Wal, 2010). It has been viewed as a core component of creative cognition (Nijstad et al., 2010; Palmiero et al., 2022). According to the dual pathway to creativity model (Nijstad et al., 2010), creative ideation relies on two distinct cognitive routes: cognitive flexibility, which involves shifting between different conceptual categories and viewpoints, and cognitive persistence, which entails systematic exploration within a single category. Although both pathways contribute to creative outcomes, flexibility is especially relevant for divergent thinking tasks that call for generating a wide range of novel responses (Palmiero et al., 2022). When individuals experience an enhanced sense of cognitive flexibility during a creative task, whether this is triggered by the energizing effect of a fast tempo or the contemplative atmosphere of a slow one, they are more prone to view themselves as capable creative agents, thereby boosting their CSE (Bandura, 1997; Tierney and Farmer, 2002). In other words, tempo may exert its influence on CSE indirectly, through its impact on the subjective feeling of cognitive flexibility while performing the task. This mediation mechanism fits well with the broader social cognitive perspective, which holds that self-efficacy beliefs are partly derived from the cognitive and affective states that individuals experience during task engagement (Bandura, 1997).

It is important to recognize that this indirect pathway may not operate consistently across all individuals. According to social cognitive theory, mastery experiences, defined as an individual’s accumulated record of prior successes and failures within a particular domain, constitute the most powerful source of self-efficacy information (Bandura, 1997). Consequently, one’s prior history with creative tasks establishes a baseline level of creative confidence, which in turn shapes how external stimuli are interpreted and integrated (Bandura, 1997; Tierney and Farmer, 2011). Attribution theory (Weiner, 1985) offers further insight by highlighting systematic individual differences in how people explain their performance outcomes. Individuals who possess extensive domain-specific experience tend to attribute their successes internally and stably, for example, by thinking “I am creative”, whereas novices are more inclined to make external and unstable attributions, such as “The music made me think faster”. This attributional tendency plays a significant role in translating task experiences into self-efficacy beliefs.

For those with a rich background of creative achievements, a solid and internalized foundation of self-efficacy has already been established (Pretz and Nelson, 2017). Such individuals may interpret transient experiences of cognitive flexibility as reinforcing evidence of their existing creative ability, thereby maintaining high CSE across different auditory conditions. In addition, highly experienced individuals are likely to possess more elaborate cognitive schemas related to creative tasks, which enable them to recognize and encode flexibility experiences as personally relevant indicators of creative competence (Sawyer et al., 2003). By contrast, for individuals who have limited prior creative exposure, the connection between cognitive flexibility and CSE may be weak or altogether absent. Without a stable internal reference point, these individuals may fail to perceive task-based cognitive experiences as diagnostic of their own creative potential, or they might attribute such flexibility to external sources, such as the music itself, rather than to their own capabilities (Bandura, 1997; Weiner, 1985). Therefore, the effect of tempo on CSE, mediated through cognitive flexibility, should be observable primarily among those with high prior creative experience, whereas those with low prior experience would be largely unaffected by tempo manipulations.

This theoretical framework gives rise to a moderated mediation model. Such a model enables researchers to move beyond simple main-effect questions and pose more targeted inquiries, namely, for whom does music tempo affect creative self-efficacy, and through what mechanism does it do so. This nuanced perspective addresses the longstanding inconsistencies in the music-and-creativity literature by specifying both the boundary conditions and the mediating processes involved. On the basis of the foregoing reasoning, the present study proposes the following hypotheses.

*H1*: Music tempo condition significantly predicts cognitive flexibility, with fast-tempo music enhancing cognitive flexibility relative to slow-tempo music.

*H2*: Cognitive flexibility positively predicts post-task CSE.

*H3*: Cognitive flexibility mediates the relationship between music tempo condition and CSE.

*H4*: Prior creative experience moderates the positive relationship between cognitive flexibility and CSE. Specifically, the flexibility-to-CSE pathway is stronger for individuals with high (vs. low) prior creative experience.

These hypotheses collectively depict a moderated mediation model (see Figure 1).

**Figure 1:**
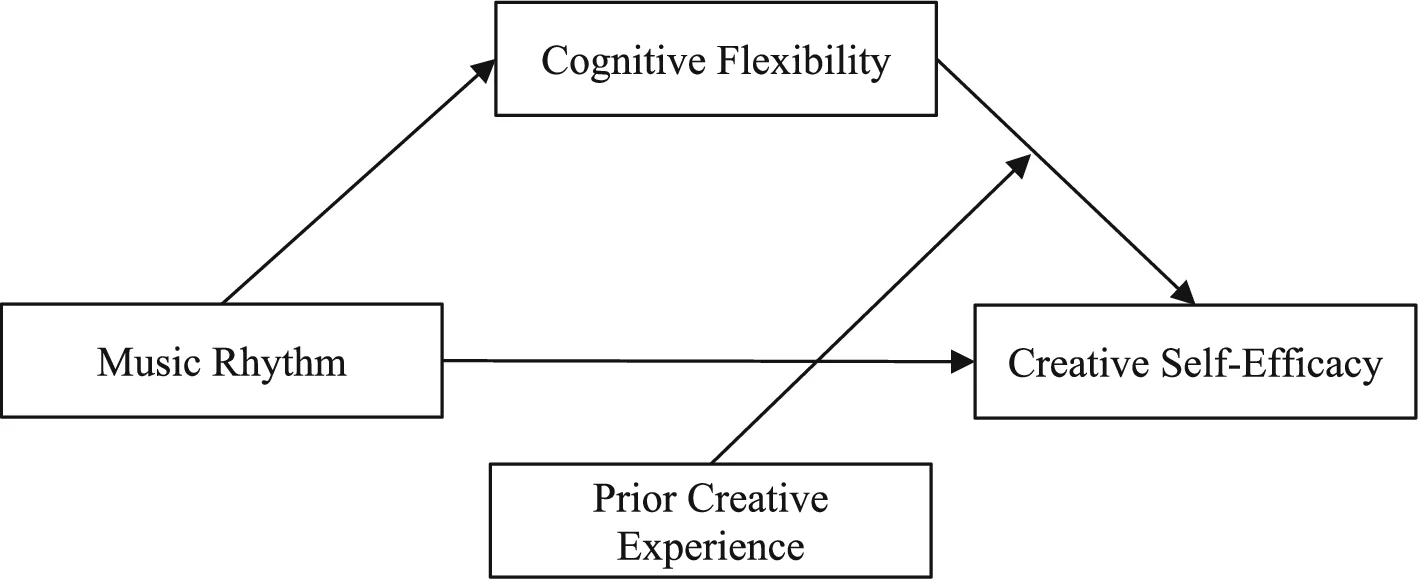
The proposed model.

## 2 Method

### 2.1 Research design and participants

The proposed moderated mediation model was tested using a three-group between-subjects experimental design. The independent variable, music condition, was manipulated at three levels: fast-tempo music, slow-tempo music, and a silent no-music control. Before the main study began, we conducted an *a priori* power analysis using G*Power 3.1 to estimate the necessary sample size. Given the expected small effect size for the interaction term (*f*^2^ = 0.02), and with alpha set at 0.05 and desired statistical power (1 – *β*) at 0.80, the analysis suggested that at least 393 participants would be required. To compensate for potential attrition or invalid responses due to inattention or technical problems, we oversampled and recruited 430 undergraduate students from a public university in China.

Following data screening, 28 individuals were removed because of incomplete questionnaires, leaving a final analytic sample of 402 participants. Their mean age was 19.95 years (SD = 1.17), ranging from 18 to 22, and the gender distribution was 51.99% female and 48.01% male. All participants had no formal musical training beyond the standard school curriculum. They were randomly assigned to one of three conditions, with 134 participants in each group (fast-tempo, slow-tempo, and control).

The research protocol received ethical approval from the Institutional Review Board of the corresponding author’s institution, and all procedures complied with the Declaration of Helsinki. Prior to participation, each individual gave written informed consent after receiving a detailed explanation of the experimental procedures, their right to withdraw at any time without penalty, and the measures in place to ensure data anonymity and confidentiality.

### 2.2 Stimuli and materials

#### 2.2.1 Auditory stimuli and tempo manipulation

To isolate the contribution of tempo structure while keeping other musical attributes, such as melody, harmony, instrumentation, timbre, familiarity, and emotional valence, as constant as possible, we adopted the sister-piece technique that is frequently used in music psychology research (Sakai et al., 2026; Hasegawa et al., 2025). In this approach, fast and slow renditions of the same composition are created by digital time-stretching, so that all structural features except tempo remain identical across conditions.

The base stimulus was a Baroque piano piece, Bach’s Minuet in G Major (BWV Anh. 114). This selection was made for several reasons. It is relatively unfamiliar to our target population, Chinese undergraduate students, which minimizes the risk of familiarity effects. The piece also has a neutral affective character and a simple harmonic structure, thus reducing extraneous emotional associations. Furthermore, its moderate original tempo allows for considerable acceleration and deceleration without producing noticeable audio artifacts.

The original tempo of the piece was 120 BPM. Two versions were generated using the Change Tempo effect in Audacity (version 3.7.1), which modifies playback speed without altering pitch or timbre. The fast-tempo condition increased the original tempo to 168 BPM (+40%), whereas the slow-tempo condition decreased it to 72 BPM (−40%). The exact BPM values for each condition were therefore 168 BPM (fast), 120 BPM (original base), and 72 BPM (slow). Both excerpts were standardized to a duration of 90 s and delivered through noise-canceling headphones at approximately 65 dB SPL. During the experimental session, the excerpt was played on a continuous loop. For the no-music control group, participants wore the same headphones but heard no sound. This procedure follows common practice in auditory distraction research, because it offers a true baseline against which the effects of music can be assessed, free from any arousal or attentional influence that non-musical auditory stimuli might introduce.

#### 2.2.2 Creative task: alternative uses task (AUT)

The Alternative Uses Task (Guilford, 1967) served as the measure of creative ideation. Participants were given 3 mins to generate as many creative and unusual uses for a common brick as they could. The AUT is a well-established and frequently employed instrument for assessing divergent thinking, which captures the central cognitive processes involved in creative idea generation. Responses were typed directly into a text box displayed on the computer screen. The three-minute limit was adopted because it is a commonly used duration in AUT research and provides a reasonable balance between encouraging ideational fluency and avoiding excessive fatigue.

AUT responses were scored along two dimensions. Fluency was indexed by the total number of non-redundant, valid responses. Originality was scored on a 0–2 scale for each response based on the statistical rarity of the response within the sample: 0 for responses given by more than 5% of participants, 1 for responses given by 2–5%, and 2 for responses given by less than 2%. A total originality score was computed by summing the originality ratings across all valid responses. Fluency and originality are the most robust and commonly reported AUT indices (Runco and Acar, 2012). This two-dimensional approach is consistent with prior research that has operationalized divergent thinking primarily through fluency and originality (e.g., Bai et al., 2021; Normann et al., 2024).

#### 2.2.3 Measures

Cognitive flexibility was assessed using the 20-item Cognitive Flexibility Inventory developed by Dennis and Vander Wal (2010), adapted to the specific task context. Participants indicated the degree to which they had experienced cognitive flexibility while generating uses for the brick (e.g., “During the task, I felt I could shift between different ways of thinking about the brick”). In the present study, the scale showed good internal reliability (Cronbach’s α = 0.87). Creative self-efficacy was measured with the three-item scale developed by Beghetto (2006). The items assess individuals’ beliefs about their creative ability (e.g., “I am good at coming up with new ideas”). In the present study, the scale showed high internal consistency (Cronbach’s *α* = 0.88). Prior creative experience was captured by a single-item measure adapted from Chua and Iyengar (2008). Participants indicated their agreement with the statement “I have considerable experience with creative tasks.” Although single-item scales are concise, we chose this approach because prior evidence indicates that such measures can have adequate predictive validity, especially when the construct is concrete and unambiguous (Bergkvist and Rossiter, 2007). All items were answered on a five-point Likert scale, with anchors ranging from 1 (strongly disagree) to 5 (strongly agree).

### 2.3 Procedure

The experimental session began with the completion of the prior creative experience measure. Afterwards, participants were randomly allocated to one of the three experimental groups. Those assigned to the two music conditions were asked to put on the headphones and adjust the playback level to a comfortable setting. They then listened to the assigned instrumental excerpt for 1 min, which allowed them to become familiar with its tempo. While they worked on the three-minute AUT, the same excerpt continued to play on a continuous loop in the background at approximately 65 dB SPL. For the no-music control group, participants wore identical headphones but received no audio input, as the channel was muted; this ensured that the physical conditions were equivalent across all groups. A countdown timer was continuously visible on the screen so that participants could monitor their own progress. However, no additional auditory or pop-up time reminders were given during the task, in order to minimize potential anxiety. Immediately after the AUT ended, the background music stopped, and participants filled out the cognitive flexibility scale, followed by the creative self-efficacy measure. The entire procedure lasted about 10 to 12 min in total.

### 2.4 Data analysis

All analyses were carried out using SPSS version 25.0. The proposed model is a moderated mediation model in which music tempo condition predicts CSE through cognitive flexibility, and prior creative experience moderates the second stage of the mediation pathway, that is, the cognitive flexibility-to-CSE relationship. In other words, the indirect effect of tempo on CSE via cognitive flexibility is specified to depend on individuals’ prior creative experience. This conceptual specification corresponds to Model 14 in the PROCESS macro. Model 14 was chosen on *a priori* theoretical grounds. Both social cognitive theory (Bandura, 1997) and attribution theory (Weiner, 1985) are consistent with the prediction that prior mastery experience should alter how task-based cognitive flexibility is translated into self-efficacy beliefs, rather than moderating the tempo-to-flexibility path or the direct tempo-to-CSE path. Specifically, individuals with greater prior creative experience should be more likely to interpret momentary cognitive flexibility as internal and stable evidence of their own creative ability, whereas those with less experience should be less likely to do so. Model 14 directly tests this prediction by estimating the conditional indirect effect of tempo on CSE through cognitive flexibility at different levels of prior creative experience. The proposed moderated mediation model was tested with the PROCESS macro version 3.0 for SPSS, specifically Model 14. To estimate the conditional indirect effects, we employed bootstrapping with 5,000 resamples, which provided bias-corrected 95% confidence intervals. In addition, separate one-way ANOVAs were conducted to examine the effects of music tempo on AUT fluency and AUT originality. These analyses tested whether the tempo manipulation was associated with the quantity or the novelty of generated ideas. *Post hoc* comparisons were conducted using Bonferroni correction.

## 3 Results

### 3.1 Descriptive statistics and correlations

Table 1 presents the means, standard deviations, and Pearson correlations among all main study variables. Cognitive flexibility was positively correlated with CSE (*r* = 0.31, *p* < 0.001), and prior creative experience was positively correlated with both cognitive flexibility (*r* = 0.14, *p* < 0.01) and CSE (*r* = 0.27, *p* < 0.001). These correlations provided preliminary support for the hypothesized relationships. Descriptive statistics for AUT fluency and AUT originality by condition are showed in Figure 2.

**Table 1:** Means, standard deviations, and correlations among main variables.

Variables	Cognitive flexibility	Creative self-efficacy	Prior creative experience	Mean (SD)
Cognitive flexibility	1	0.31***	0.14**	2.98 (0.45)
Creative self-efficacy		1	0.27***	2.94 (0.76)
Prior creative experience			1	3.22 (0.91)

^***^ *p* < 0.001, ** *p* < 0.01.

**Figure 2:**
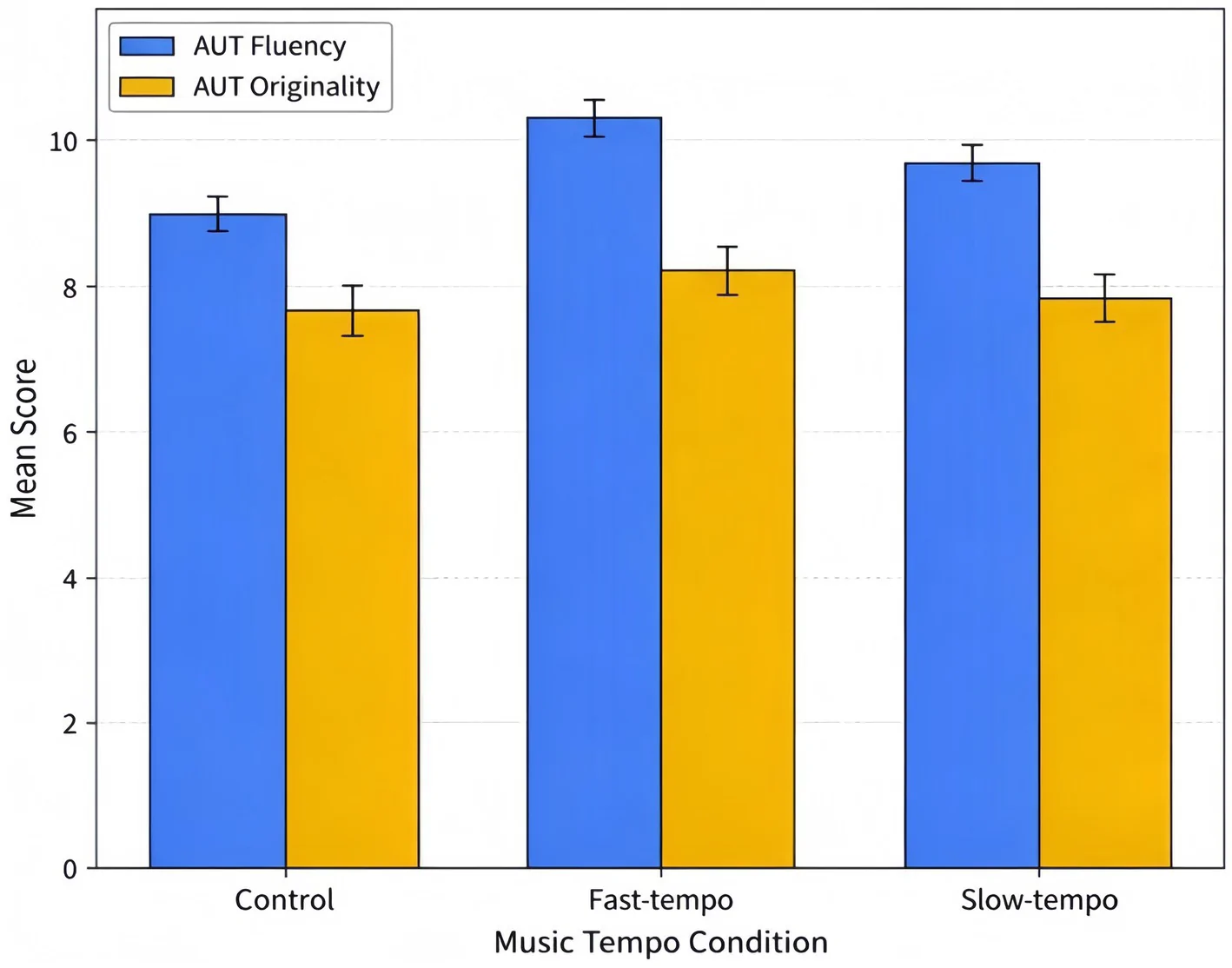
Mean scores on AUT fluency and originality across the fast-tempo, slow-tempo, and control conditions. Error bars represent standard errors.

### 3.2 Testing the moderated mediation model

The mediator model regressed cognitive flexibility on the two dummy-coded music condition variables. The overall model was significant, *R*^2^ = 0.039, *F* = 3.99, *p* < 0.01. As shown in Table 2, the fast-tempo condition significantly predicted higher cognitive flexibility compared to the control condition (*B* = 0.20, *p* < 0.001, 95% CI [0.10, 0.31]), supporting Hypothesis 1 for the fast-tempo contrast. In contrast, the slow-tempo condition did not significantly differ from the control condition (*B* = 0.08, *n.s*., 95% CI [−0.03, 0.18]). These results indicated that the fast-tempo music enhanced cognitive flexibility relative to the silent baseline.

**Table 2:** Regression results for mediator model (DV: cognitive flexibility).

Predictor	*B*	SE	*t*	*p*	LLCI	ULCI
Constant	−0.43	0.38	−1.12	0.264	−1.18	0.32
age	0.02	0.02	0.82	0.411	−0.02	0.05
gender	0.04	0.04	0.92	0.360	−0.05	0.13
Fast-tempo (D1)	0.20	0.05	3.74	<0.001	0.10	0.31
Slow-tempo (D2)	0.08	0.05	1.42	0.157	−0.03	0.18

D1 = fast-tempo condition (1 = fast, 0 = other); D2 = slow-tempo condition (1 = slow, 0 = other); LLCI, lower level 95% confidence interval; ULCI, upper level 95% confidence interval.

The dependent variable model regressed CSE on the two dummy-coded music condition variables, cognitive flexibility, prior creative experience, and the interaction term. The overall model was significant, *R*^2^ = 0.172, *F* = 11.68, *p* < 0.001. Table 3 shows that cognitive flexibility was a positive predictor of CSE (*B* = 0.50, *p* < 0.001, 95% CI [0.35, 0.66]), supporting Hypothesis 2. The indirect effect of fast-tempo (relative to control) on CSE via cognitive flexibility was significant (effect = 0.10, *SE* = 0.03, 95% CI [0.04, 0.17]). For the slow-tempo contrast, however, the indirect effect was not significant (effect = 0.04, *SE* = 0.03, 95% CI [−0.02, 0.10]).

**Table 3:** Regression results for dependent variable model (DV: Creative self-efficacy).

Predictor	*B*	SE	*t*	*p*	LLCI	ULCI
Constant	3.11	0.60	5.19	<0.001	1.93	4.29
age	−0.01	0.03	−0.30	0.768	−0.07	0.05
gender	−0.02	0.07	−0.31	0.755	−0.15	0.12
Fast-tempo (D1)	−0.03	0.09	−0.39	0.702	−0.20	0.14
Slow-tempo (D2)	0.02	0.09	0.275	0.783	−0.14	0.19
Cognitive flexibility	0.50	0.08	6.29	<0.001	0.35	0.66
Prior creative experience	0.18	0.04	4.76	<0.001	0.11	0.26
CF × PCE	0.31	0.09	3.40	<0.001	0.13	0.48

D1 = fast-tempo condition (1 = fast, 0 = other); D2 = slow-tempo condition (1 = slow, 0 = other); CF, Cognitive flexibility; PCE, Prior creative experience; LLCI, lower level 95% confidence interval; ULCI, upper level 95% confidence interval.

More importantly, the interaction between cognitive flexibility and prior creative experience was significant (*B* = 0.31, *p* < 0.001, 95% CI [0.13, 0.48]), thereby supporting Hypothesis 4. This interaction indicated that the positive relationship between cognitive flexibility and CSE was stronger for individuals with higher levels of prior creative experience. Notably, the direct effects of both music conditions on CSE were not significant after accounting for the mediator and the interaction (fast-tempo: *B* = −0.03, *n.s.,* 95% CI [−0.20, 0.14]; slow-tempo: *B* = 0.02, *n.s*., 95% CI [−0.14, 0.19]). To further probe the significant interaction between cognitive flexibility and prior creative experience, we conducted a simple slopes analysis (see Figure 3). At high levels of prior creative experience (+1 SD), the relationship between cognitive flexibility and CSE was strong and significant (*B* = 0.78, *p* < 0.001). At low levels of prior experience (−1 SD), the relationship was substantially weaker but remained significant (*B* = 0.22, *p* < 0.05). This pattern indicated that while cognitive flexibility consistently predicted CSE across all levels of prior experience, the strength of this association increased with prior creative experience.

**Figure 3:**
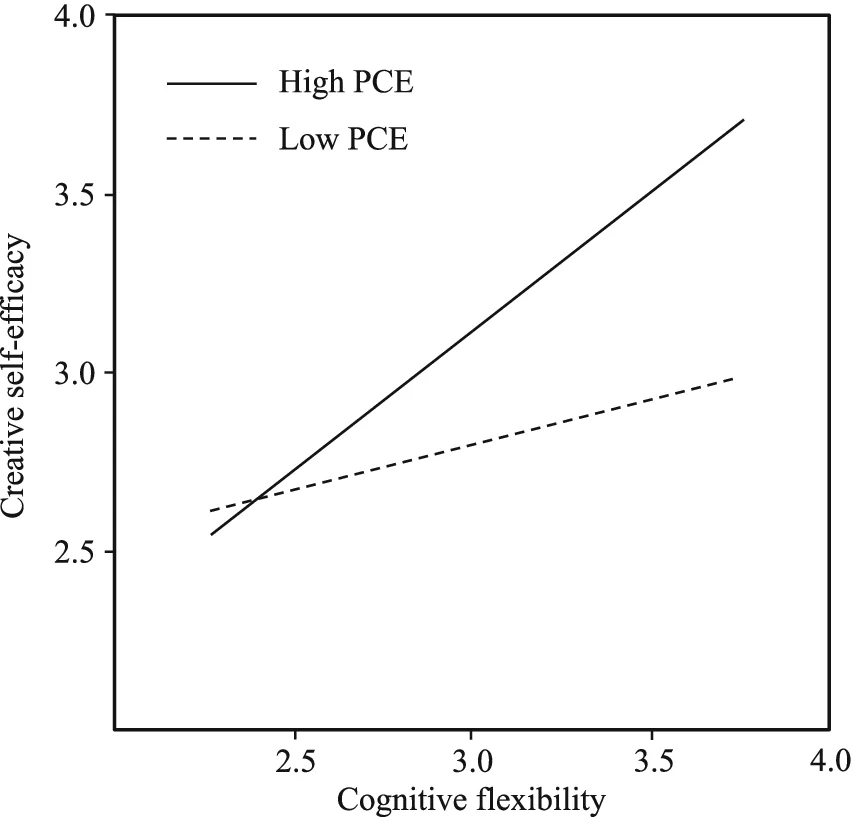
The effect of cognitive flexibility on creative self-efficacy at different levels of prior creative experience.

The conditional indirect effects are summarized in Table 4. For the fast-tempo contrast, the indirect effect was significant for individuals with high prior experience (effect = 0.16, 95% CI [0.07, 0.27]), but not significant for individuals with low prior experience (effect = 0.04, 95% CI [−0.00, 0.10]). The index of moderated mediation was significant for the fast-tempo contrast (effect = 0.06, 95% CI [0.02, 0.12]), confirming that the indirect effect significantly varied across levels of prior creative experience. For the slow-tempo contrast, neither the conditional indirect effects nor the index of moderated mediation reached significance.

**Table 4:** Conditional indirect effects of tempo condition on CSE via cognitive flexibility at different levels of prior experience.

Contrast	PCE level	Effect	Boot SE	BootLLCI	BootULCI
Fast vs. Control	Low (−1 SD)	0.04	0.03	0.00	0.10
	High (+1 SD)	0.16	0.05	0.07	0.27
	Index of moderated mediation	0.06	0.03	0.02	0.12
Slow vs. Control	Low (−1 SD)	0.02	0.02	−0.01	0.06
	High (+1 SD)	0.06	0.04	−0.02	0.15
	Index of moderated mediation	0.02	0.02	−0.01	0.07

Bootstrapping with 5,000 resamples. LLCI, lower level 95% confidence interval; ULCI, upper level 95% confidence interval; PCE, Prior experience levels.

### 3.3 Effects of music tempo on AUT fluency and originality

Separate one-way ANOVAs were conducted to explore whether music tempo condition affected AUT fluency and originality. For fluency, the effect of condition was significant, *F*(2, 399) = 6.95, *p* < 0.01, η^2^ = 0.034. Bonferroni-corrected *post hoc* comparisons indicated that participants in the fast-tempo condition (*M* = 10.29, SD = 2.98) generated significantly more valid responses than those in the control condition (*M* = 8.98, SD = 2.74, *p* < 0.001, 95% CI [0.63, 2.00]), whereas the slow-tempo condition (*M* = 9.67, SD = 2.93) did not differ significantly from either the fast-tempo condition (*n.s*., 95% CI [−0.09, 1.33]) or the control condition (*n.s.,* 95% CI [−0.01, 1.37]).

For originality, the effect of condition was not significant, *F*(2, 399) = 0.67, *n.s*., η^2^ = 0.003. The mean originality scores were 8.21 (SD = 3.85) in the fast-tempo condition, 7.84 (SD = 3.76) in the slow-tempo condition, and 7.67 (SD = 4.03) in the control condition. These results indicate that although fast-tempo music was associated with greater fluency, it was not associated with higher originality. This pattern suggests that tempo may primarily facilitate the quantity or speed of idea generation rather than the novelty of the ideas produced.

Taken together, the results from the moderated mediation model and the exploratory AUT analyses suggest that music tempo influenced the subjective experience of cognitive flexibility and, indirectly, CSE, while its behavioral effect was limited to the fluency of idea generation. The absence of a significant tempo effect on originality indicates that the observed enhancement in creative self-efficacy should not be interpreted as an enhancement in the quality or novelty of creative output.

## 4 Discussion

The present research was designed to test whether and under what conditions music tempo influences CSE. Our theoretical integration combined social cognitive theory, the dual pathway model, and attribution principles, leading us to expect that tempo would operate indirectly through cognitive flexibility, and that this indirect pathway would be gated by prior creative experience. The results supported this expectation. Fast-tempo music significantly raised cognitive flexibility compared to silence, while slow-tempo music did not. Cognitive flexibility, in turn, positively predicted CSE. However, the indirect effect via cognitive flexibility was not uniform across participants. It reached significance for those with a strong history of creative mastery, but not for those with little prior creative exposure. Thus, our study not only identifies cognitive flexibility as a key transmitting mechanism, but also specifies a crucial individual difference that determines whether the auditory manipulation actually affects self-efficacy. This dual insight may help resolve the longstanding empirical ambiguity surrounding music’s role in creative cognition.

### 4.1 The differential effect of tempo on cognitive flexibility

The observed asymmetry between fast-tempo and slow-tempo conditions is consistent with the broader literature on temporal entrainment. Research has shown that rapid temporal pulses elevate physiological arousal, sharpen attentional orienting, and promote neural synchronization in frequency bands relevant to cognitive control and flexible updating of information (Doelling and Poeppel, 2015; Thaut et al., 2015). Within the domain of divergent thinking, this combination of heightened arousal and enhanced temporal predictability may reduce the threshold for associative retrieval, thereby facilitating shifts between conceptual categories, a process widely recognized as central to cognitive flexibility (Nijstad et al., 2010; Palmiero et al., 2022). The non-significant slow-tempo effect relative to silence may indicate that a decelerated beat does not provide sufficient energetic or temporal scaffolding to elevate flexibility above baseline. This asymmetric pattern is consistent with prior observations that tempo effects on cognition are often nonlinear, with moderate to fast tempi preferentially supporting speeded or associative processing and slower tempi tending to favor reflective or convergent modes of thought (De Francesco et al., 2025; Liu, 2026). Importantly, our use of time-stretched sister pieces, which held melody, harmony, and timbre constant across conditions, enabled us to isolate tempo from other musical features and thereby to reduce confounds that have complicated earlier work (Sun et al., 2024; Threadgold et al., 2019). The present results therefore offer relatively unambiguous evidence that tempo alone, and especially a fast tempo, can facilitate cognitive flexibility.

It is important to acknowledge, however, that the effect size of tempo alone was small. The fast-tempo condition explained only a modest portion of variance in cognitive flexibility (R^2^ = 0.039), and the direct effects of music condition on CSE were non-significant. This is consistent with the broader literature indicating that background music, by itself, is a weak environmental influence on creative cognition (de la Mora Velasco et al., 2023). The more substantial finding of the present study is that the indirect effect of tempo on CSE became considerably larger when intra-individual characteristics, specifically, prior creative experience, were taken into account. The index of moderated mediation was significant. This suggests that the psychological impact of auditory environmental cues is not primarily determined by the stimulus itself, but by the extent to which individuals possess the experiential resources needed to interpret transient cognitive states as self-relevant evidence of creative ability. In other words, tempo alone may be a weak agent of change, but it can become a meaningful catalyst when it interacts with a well-developed creative self-schema. This conclusion aligns with the social cognitive view that self-efficacy beliefs are shaped by the interplay between external cues and internal mastery histories (Bandura, 1997), and it underscores the value of moving beyond main-effect designs in research on music and creativity.

### 4.2 The mediating role of cognitive flexibility and the moderating role of prior creative experience

The indirect effect of fast-tempo music on CSE via cognitive flexibility reached significance, supporting the proposed mediation hypothesis. This pattern is consonant with Bandura's (1997) social cognitive framework, which holds that self-efficacy beliefs draw partly on the cognitive and affective states individuals experience while performing a task. Participants who experienced heightened cognitive flexibility in response to the fast tempo translated that subjective state into enhanced creative confidence. This finding extends prior meta-analytic evidence that CSE is sensitive to proximal task experiences (Haase et al., 2018; Tierney and Farmer, 2011) by showing that even a brief auditory manipulation can alter such experiences.

The moderated mediation analysis further revealed that this indirect pathway operated among participants with high prior creative experience; for participants with low prior creative experience, the indirect effect was not statistically significant. This pattern is consistent with two complementary theoretical accounts. Bandura (1997) identified mastery experiences as the foremost source of self-efficacy, whereas Weiner (1985) emphasized that individuals differ systematically in how they attribute performance outcomes. For those with extensive creative histories, experiences of cognitive flexibility are likely to be perceived as internal and stable evidence of creative ability. Such individuals also possess more elaborated cognitive schemas for creative tasks, enabling them to encode momentary flexibility as personally relevant feedback (Sawyer et al., 2003). Novices, by contrast, lack a stable internal reference point. They may attribute enhanced flexibility to external sources, such as the music itself, or fail to recognize such experiences as diagnostic of their own capabilities, thus blocking the transfer from flexibility to self-efficacy. The simple slopes analysis reinforces this interpretation. Although cognitive flexibility predicted CSE positively at both high and low levels of prior experience, the association was markedly stronger for the high-experience group, indicating that prior mastery amplifies the psychological value of cognitive flexibility.

The exploratory AUT analyses add an important qualification to this interpretation. Fast-tempo music was associated with higher fluency but not with significantly higher originality. This dissociation suggests that a faster tempo may primarily spur faster idea generation rather than deeper or more novel ideation. Such a pattern is theoretically meaningful: it implies that tempo may be more useful at the generative, idea-production stage of the creative process than at later stages that require elaboration, evaluation, or refinement. From a practical standpoint, this means that fast-tempo music should not be treated as a general-purpose creativity enhancer; its benefits may be stage-specific. It also raises the possibility that different tempi might support different phases of the creative process, with faster tempi facilitating initial ideation and slower tempi or silence supporting deeper elaboration and convergent thinking.

These observations also resonate with the work of Fritz et al. (2020), who showed that physical exercise combined with musical feedback increased creativity in a divergent thinking task more than either musical control or physical exercise alone. Their finding indicates that arousal alone, or direct creative expression alone, may not be sufficient to induce creativity; rather, creativity may be enhanced when multiple channels (physiological arousal and musical engagement) are combined. Our results are consistent with this view. Fast tempo alone produced only a small effect on cognitive flexibility and did not directly predict CSE. It was when prior creative experience was taken into account that the indirect pathway became significant. This pattern supports the idea that tempo-based arousal is not automatically beneficial; its effects depend on whether individuals can integrate the aroused state into an existing creative self-schema. In this sense, the current study complements Fritz et al. (2020) by showing that the boundary condition for tempo effects may lie not only in the multimodal nature of the intervention but also in the individual’s prior mastery history.

Several observations about the direct effects deserve mention. Once the mediator and the interaction were accounted for, the direct paths from music condition to CSE were non-significant. This pattern is consistent with full mediation for the fast-tempo contrast within the high-experience subgroup, although non-significant direct effects should be interpreted with caution. This specificity offers a refinement to theoretical models that have typically treated background music as a global mood or arousal manipulator (Ritter and Ferguson, 2017) and highlights the need to disentangle cognitive mechanisms from affective ones.

### 4.3 Theoretical and practical implications

This study offers several theoretical implications. First, the dual pathway to creativity model (Nijstad et al., 2010) has typically been applied to performance outcomes; our findings extend it by identifying music tempo as an environmental factor that selectively activates the cognitive flexibility route, and by linking this route to a motivational outcome, namely CSE, rather than to creative performance alone. Second, we contribute to social cognitive theory by specifying how external auditory cues interact with internal mastery histories in shaping self-efficacy beliefs. Previous applications of this framework have emphasized social persuasion, vicarious experience, and affective states, but have given less attention to how environmental sensory inputs combine with personal history. Our moderated mediation model fills this gap. Third, the study introduces attribution theory (Weiner, 1985) as a complementary explanatory mechanism for the moderating role of prior experience, thereby offering a fresh perspective for future investigations into self-perception in creative domains.

On the practical side, these findings carry implications for educational and organizational settings where background music is frequently used. For individuals with extensive creative expertise, including experienced designers, writers, or scientists, fast-tempo instrumental music may serve as a simple and inexpensive means to reinforce creative confidence during ideation. For novices or students at early stages of creative development, however, the same auditory environment is unlikely to produce the intended motivational benefit; relying on music alone would be ineffective. The small effect size of tempo alone further cautions against overestimating the power of background music as a standalone intervention.

Educators and managers should therefore assess the learner’s or employee’s experiential background before implementing auditory interventions, and should consider combining music with other supports, such as structured creative training or mastery-building experiences, rather than relying on music in isolation. More broadly, our results caution against uniform recommendations about background music and point toward the need for personalized or adaptive sound environments tailored to individual experience levels.

### 4.4 Limitations and future directions

Several methodological considerations warrant attention when interpreting these findings. The first concerns sample composition. Our participants were all Chinese undergraduates, a demographic that, while reducing extraneous variability and thereby strengthening internal validity, may limit the generalisability of the results to other age groups, educational levels, or cultural settings. Cross-sample replication is therefore needed to establish whether the moderated mediation pattern is culturally universal or context-dependent. A second issue relates to measurement. Prior creative experience was assessed with a single self-report item. Although such measures have shown acceptable predictive validity for concrete constructs (Bergkvist and Rossiter, 2007), multi-item instruments that capture both the breadth and depth of creative achievements would provide a more nuanced assessment and might reveal finer-grained moderating effects. Furthermore, the Alternative Uses Task, despite its status as a well-validated measure of divergent thinking, taps only one dimension of creativity. Whether the observed effects extend to convergent thinking, artistic production, or complex real-world problem-solving remains an open question for future investigation. A related limitation concerns ecological validity. The use of a Baroque piano piece, though methodologically necessary to isolate tempo through the sister-piece technique, raises questions about generalisability to naturalistic creative environments. For our Chinese sample, culturally familiar music, such as traditional guqin pieces, might produce different patterns of response. Future studies using indigenous auditory stimuli are therefore warranted. Fourth, although the AUT was scored for fluency and originality, we did not score flexibility or elaboration. This limits the conclusions that can be drawn about the qualitative diversity and depth of creative ideas. Future studies should include the full four-dimension scoring to examine whether fast tempo selectively increases idea quantity and novelty while leaving flexibility or elaboration unaffected.

Taken together, these findings establish that fast-tempo music can enhance creative self-efficacy indirectly through cognitive flexibility among individuals with a substantial history of creative mastery; for those with low prior creative experience, the indirect effect was not statistically significant. By identifying both the mediating mechanism and the moderating boundary condition, our study provides a theoretically grounded framework that helps reconcile long-standing inconsistencies in the literature. These results also highlight the importance of tailoring auditory interventions to individual differences and point to the value of continued research on how external sensory inputs, cognitive states, and self-perceptual processes interact in the domain of creativity.

## Data Availability

The original contributions presented in the study are included in the article/Supplementary material, further inquiries can be directed to the corresponding author.
